# MicroRNAs and Drug Resistance in Non-Small Cell Lung Cancer: Where Are We Now and Where Are We Going

**DOI:** 10.3390/cancers14235731

**Published:** 2022-11-22

**Authors:** Roberto Cuttano, Miriam Kuku Afanga, Fabrizio Bianchi

**Affiliations:** Unit of Cancer Biomarkers, Fondazione IRCCS Casa Sollievo della Sofferenza, 71013 San Giovanni Rotondo, Italy

**Keywords:** non-small cell lung cancer, NSCLC, microRNAs, gene expression, drug resistance

## Abstract

**Simple Summary:**

The problem of drug resistance represents a major challenge for the cure of non-small cell lung cancer. Tumor cells can be intrinsically resistant to drugs or they can become resistant during the treatment as a result of several adaptive responses. However, molecular mechanisms at the basis of drug resistance are not fully understood. MicroRNAs are small non-coding RNA that regulate gene expression and play critical functions in many cellular processes. Recently, the study of microRNAs has provided evidence about their role in the regulation of molecular mechanisms at the basis of drug resistance. Here we summarize the available knowledge about the role of miRNAs in the resistance to drugs that are currently used to treat non-small cell lung cancer and we critically discuss the experimental approaches on the basis of this evidence.

**Abstract:**

Lung cancer is the leading cause of cancer-related mortality in the world. The development of drug resistance represents a major challenge for the clinical management of patients. In the last years, microRNAs have emerged as critical modulators of anticancer therapy response. Here, we make a critical appraisal of the literature available on the role of miRNAs in the regulation of drug resistance in non-small cell lung cancer (NSCLC). We performed a comprehensive annotation of miRNAs expression profiles in chemoresistant versus sensitive NSCLC, of the drug resistance mechanisms tuned up by miRNAs, and of the relative experimental evidence in support of these. Furthermore, we described the pros and cons of experimental approaches used to investigate miRNAs in the context of therapeutic resistance, to highlight potential limitations which should be overcome to translate experimental evidence into practice ultimately improving NSCLC therapy.

## 1. Introduction

Lung cancer represents the main cause of cancer-related death worldwide. Despite recent advances in lung cancer management, the prognosis of patients remains adverse with five-year survival rates ranging from 6% in the case of metastatic cancer (i.e., regional and distant metastases) up to 33–60% in patients with localized tumours [1]. Non-small-cell lung cancer (NSCLC) is the most common subtype of lung cancer (i.e., 85% of cases; [2]). Therapeutic approaches for the treatment of NSCLC include surgery, radiotherapy, chemotherapy, targeted therapy, and immunotherapy used alone or in combination. Although the number of therapeutic agents for NSCLC treatment has dramatically increased in the last years, a long-term impact in the setting of advanced disease has not been reached due to the acquisition of drug resistance [3]. Thus, a better understanding of the mechanisms underlying the response to therapy is needed to achieve long-term and durable disease remission. In this regard, the investigation of the coding and non-coding transcriptome (e.g., microRNAs [miRNAs]) of NSCLC in the last years has led to important findings on which and how specific molecular mechanisms are involved in drug resistance. Indeed, several studies which focused on miRNA expression profiling in NSCLC have shown their frequent altered expression pattern in lung cancer [4,5,6] and their role as master regulators of cancer genes involved in chemotherapy response [7].

MiRNAs are non-coding RNAs (ncRNAs) of ~22 nucleotides, which act mainly as post-transcriptional gene regulators [8]. The impact of miRNAs in the regulation of the transcription of genes can be relevant since one miRNA can potentially target the messenger RNAs (mRNAs) of distinct genes. Yet, the expression of a single gene can be regulated by different miRNAs containing a similar seed sequence (i.e., a conserved heptametrical sequence which is mostly situated at positions 2–7 from the miRNA 5′-end) which, ultimately, targets the relative complementary sequence in the mRNA, i.e., the miRNA recognition elements (MREs). miRNA can exert other non-canonical functions, such as activating Toll-like receptors that are involved in the activation of innate immune response, binding non-argonaute (AGO) proteins (e.g., hnRNPE2) and triggering transcription, binding other ncRNAs which inhibit miRNA functions (i.e., miRNA sponges) [9]. Remarkably, miRNAs can be released in the extracellular space within extracellular vesicles (e.g., exosomes) [10] or bound to proteins such as AGO2 [11,12] and lipids [13]. These cell-free miRNAs can be then internalized by receiving cells even at a considerable distance and regulate their transcriptomes, thus acting as hormone-like molecules in an autocrine and paracrine fashion [14].

Such molecular and biological features of miRNAs make them play key roles in the regulation of physiological and pathogenic cellular processes relevant to cancer progression and therapy response.

Here, we reviewed the most relevant literature focused on the role of miRNAs and their molecular mechanisms at the basis of drug resistance in NSCLC. The main experimental strategies as well as their pros and cons were also discussed.

## 2. Relevant Sections

### 2.1. Annotation of miRNAs with a Functional Role in NSCLC Therapy Resistance

**Figure 1:**
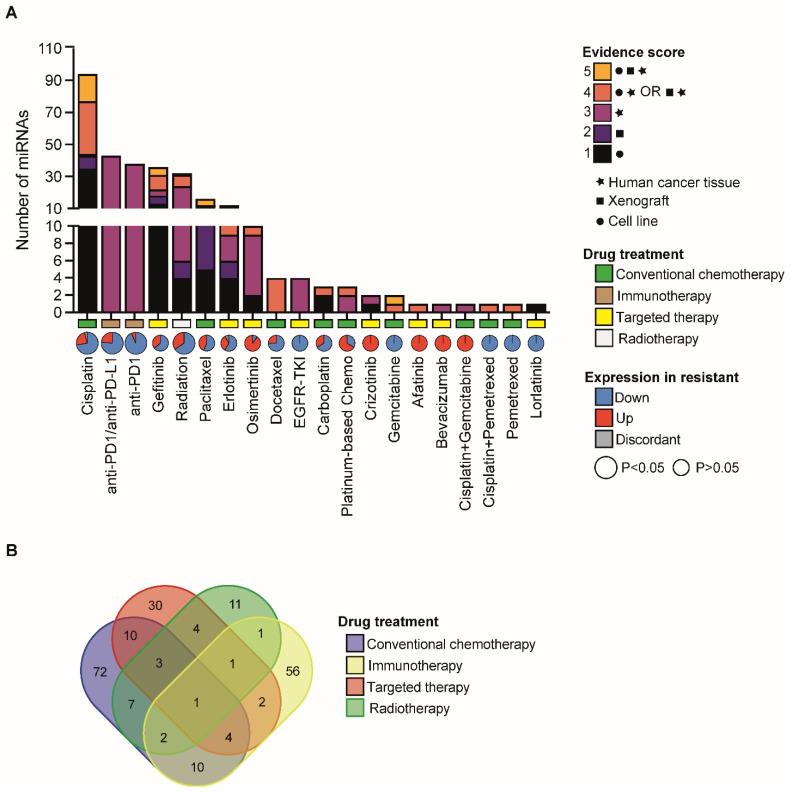
Landscape of miRNAs associated with therapy response in NSCLC. (**A**) Bar chart reporting the number of miRNAs (listed in Table S2) that are associated with therapy response in NSCLC. Different colours indicate relative evidence scores which were assigned to miRNAs based on the type of experiment conducted (i.e., cell lines, xenograft models and/or human cancer tissue). In X-axes, pie charts indicate the fraction of miRNAs whose expression is upregulated (red) or downregulated (blue) in resistant samples vs. sensitive ones. In grey, fraction of miRNAs with a discordant trend of expression regulation (up or down) in independent studies. Pie sizes are proportional to the statistical significance (binomial test) relative to the unbalance in up- or downregulated miRNAs, as per the legend. Type of drug treatment is also indicated in X-axes, as per the legend (see also Supplementary Methods for further details). (**B**) Venn diagram of the overlapping miRNAs associated to the indicated category of drug treatment. The list of miRNAs can be found in Table S3.

We initially performed a literature search (see Methods) and reviewed a total of 502 publications (from 2009 to June 2022) that report signatures of miRNAs involved in the responses to different drugs used to treat NSCLC patients (Table S1; Supplementary Methods). Next, we analyzed miRNA expression regulation in the drug-resistant condition and the various experimental models/cohort of samples (Figure 1). Furthermore, we assigned an ascending score depending on whether the role of miRNAs was investigated in cell lines (1), in animal models (2), human cancer tissues (3), cell lines AND human cancer tissues OR animal models AND human cancer tissues (4), cell lines AND animal models AND human cancer tissues (5). Importantly, this score describes the strength of the evidence supporting the role of particular miRNAs in drug resistance and can be used to select a miRNA based on the level of evidence (Figure 1; Table S2; Supplementary Methods). When available, we also reported genes that were (i) experimentally validated as a direct target of the indicated miRNA, and (ii) functionally involved in miRNA-mediated regulation of drug resistance (Table S2; Supplementary Methods). Finally, each miRNA entry on the database was associated with the relative citation by reporting the Pubmed ID (PMID) of the publication, thus allowing fast retrieval of the study of interest (Table S2; Supplementary Methods). In the case of multiple independent reports about a single miRNA, we merged the information derived from all the studies to obtain a unique miRNA entry.

### 2.2. The Landscape of miRNAs Which Regulate Cisplatin Resistance in NSCLC

Out of a total of 42 drugs used in the NSCLC clinical setting (Table S1), we found that the expression/activity of a total of 212 unique miRNAs was associated with sensitivity to 22 drugs (Figure 1; Table S2). Cisplatin emerged as the top drug whose sensitivity can be functionally modulated by miRNAs and showed the highest number of miRNAs with the strongest evidence score (Figure 1). This could be partly explained by an earlier approval (in 1978) of Cisplatin for lung cancer treatment by US food and drug administration (FDA) in comparison with more recently approved drugs [15]. Interestingly, we noticed that the resistance to cisplatin was generally and significantly associated with an overall loss of miRNA expression (Figure 1A), in line with the prevalent role of miRNAs as tumor suppressors [16]. Similarly, we observed a frequent miRNA downregulation in tumors refractory to radiotherapy and immunotherapy (Figure 1A). Notably, there is a large fraction of miRNAs involved in mechanisms of resistance to conventional chemotherapy, radio, and immunotherapy, which suggests a global transcriptome reprogramming that impacts molecular mechanisms at multiple levels (Figure 1A). Contrariwise, the number of miRNAs involved in mechanisms of resistance to targeted agents is lower and with a more heterogeneous trend of the regulation (Figure 1A), which is reasonable in the case of targeted agents that act by inhibiting/activating specific molecular mechanisms to kill tumor cells [17]. Interestingly, we found that a number of miRNAs were commonly altered in response to drugs belonging to different categories (Figure 1B; Table S3), which suggests common regulatory pathways to be involved in drug resistance. Indeed, as we previously discussed, several of these miRNAs have been already reported to regulate common pathways/signaling cascades (Table 1).

Over the last 30 years of research, a variety of molecular mechanisms were described to be involved in the onset of resistance to cisplatin which were extensively reviewed elsewhere [18]. Yet, a recent review reported a large number of genes (~900) to be implicated in platinum response in several human malignancies [19]. Mechanisms of cisplatin resistance in tumor cells can be summarized in those involved in (i) cisplatin import/export, (ii) DNA damage repair, (iii) transcriptome reprogramming by sequestering miRNAs (miRNA sponges), (iv) pro-survival and apoptotic signaling pathways, (v) oxidative stress, (vi) cancer signalling pathways, (vii) epithelial-to-mesenchymal transition (EMT), and (viii) autophagy. Here below, we provide a schematic report:

− Cisplatin import/export: regulation of cisplatin import/export is an important mechanism of resistance to cisplatin. MiR-369-3p overexpression promoted cisplatin resistance by direct regulation of the expression of the SLC35F5 gene, a nucleotide sugar transporter involved in drug uptake [20]. In addition, miR-495-3p regulates cisplatin resistance by modulating the expression of ATP7A, a copper transporter that regulate cisplatin efflux from the cells [21].
− DNA damage repair: upon entering the cells, cisplatin becomes activated by the displacement of chloride atoms by water molecules. Active cisplatin exerts its cytotoxic function mainly by generating monoadducts and crosslinks at the level of the DNA. Therefore, genetic and epigenetic alterations of genes involved in DNA damage response represent a major mechanism to cope with cisplatin-induced cell death. In line with this, miR-92a-3p regulates cisplatin resistance by directly binding to the 3′ untranslated region (UTR) of RAD21 mRNA, a member of the cohesin complex that can promote DNA repair at the G2 phase of the cell cycle [22]. Yet, miR-17-5p, which belongs to the same cluster of miR-92a-3p (e.g., miR-17-92 cluster), can protect cancer cells from cisplatin-induced apoptosis by regulating CDKN1A, a cell cycle inhibitor that blocks DNA synthesis by G1 arrest and whose levels are increased upon accumulation of DNA damage due to activation of ATM and ATR and consequent TP53 stabilization [22].
− miRNA sponges: long non-coding RNAs (lncRNAs) are transcripts longer than 200 nucleotides that do not code for functional proteins. Since lncRNAs can contain MREs in their sequence, they can sequester miRNAs and impair their activity on target genes, therefore, acting as competitive endogenous RNAs or miRNA ‘sponges’. Over the last years, a high number of reports described lncRNAs that regulate cisplatin resistance by sponging specific miRNAs. For example, NORAD is a lncRNA upregulated by DNA damage and was found involved in cisplatin resistance by regulating SOX4 expression by sponging miR-129-1-3p [23]. Similarly, circular RNA (circRNA) Circ-PRMT5 promoted the resistance to cisplatin by competing with the binding of miR-4458 to REV3L [24], a catalytic subunit of DNA polymerase implicated in the tolerance of DNA adduct through translesion synthesis.
− Pro-survival and apoptotic signaling pathways: when cells are exposed to platinum, both pro-survival and apoptotic signaling pathways are activated and compete for the final fate of the cells. A number of miRNAs have been associated with the direct or indirect regulation of apoptotic proteins or survival signaling pathways. MiR-103a-3p induces ERK signaling in NSCLC cells by targeting NF1 expression, a key negative regulator of the Ras signaling pathway [25]. In an independent study, Wang et al., found that miR-103a-3p could be released in an exosome (nanosized extracellular vesicles actively released by a variety of cells) from cancer-associated fibroblast and can induce resistance of NSCLC cells via direct regulation of BAK1, a pro-apoptotic BCL-2 family member [26]. Yet, miR-29c-3p affects cisplatin resistance by regulating PI3K/AKT signaling pathways due to binding to 3′UTR of AKT2 [27]. Similarly, miR-126-5p overexpression increases cisplatin sensitivity by inhibiting the PTEN/PI3K/AKT signaling pathway, an effect partly induced by the direct regulation of the metalloprotease ADAM9 [28]. In addition, miR-539-5p increases the sensitivity of cisplatin-resistant cells via the inactivation of the P13K/AKT/mTOR signaling pathway by targeting the protein kinase DCLK1 [29]. Interestingly, the increase in DCLK1 expression and cisplatin resistance was found to be also mediated by lncRNA SNHG1-dependent sponging of miR-330-5p [30]. Several independent studies associated also miR-21 upregulation with increased resistance to Cisplatin [31,32,33,34,35,36,37], mostly due to a direct regulation of PTEN expression [32,33,35,36]. Furthermore, miR-21 expression was reported to be regulated by KRAS wild-type or mutant [37], a major driver mutation in NSCLC.
− Reactive oxygen species: besides DNA damage, activated cisplatin is also a potent inducer of reactive oxygen species, which induces cell death. In this scenario, miR-495-3p overexpression was reported to modulate cisplatin resistance through direct inhibition of NRF2 [38], a transcription factor that regulates the expression of important NADPH-generating enzymes and redox proteins crucial for protecting the cells from oxidative stress.
− Epithelial-to-mesenchymal transition (EMT): EMT contributes to resistance to several therapeutic agents, including cisplatin [39]. In line with this, miR-128-3p upregulation drives chemoresistance and was associated with the overactivation of Wnt/beta-catenin and TGF-beta (TGF-β) pathways and consequent acquisition of mesenchymal and stem-like features [40]. Likewise, miR-181b-5p regulates the TGF-β pathway by direct inhibition of TGFBR1 expression, thus modulating EMT and sensitivity to cisplatin [41]. An independent study found that miR-181b-5p suppresses stem cell properties in tumor cells and enhances sensitivity to cisplatin treatment by directly targeting NOTCH [42].
− Autophagy: autophagy is a crucial process that allows the recycling of important cellular components in response to stress conditions such as those induced by cisplatin treatment. Indeed, regulation of autophagy has been widely associated with cisplatin resistance phenotype. Rescue of miR-1-3p increases the sensitivity of cisplatin resistance cells by inhibiting ATG3, a key autophagic protein [43]. Furthermore, exosomal transfer of miR-425-3p was found to increase autophagic flux and chemoresistance by inhibiting AKT1 in the targeted NSCLC cells [44].

### 2.3. Beyond Targeting NSCLC Cells: The Role of miRNA in Regulating Immune Response to Cisplatin Treatment

Besides targeting tumor cells, platinum compounds can also elicit an immune response against cancer cells through different mechanisms, i.e., by augmenting CD8^+^ T cells tumor infiltration, inducing maturation of Antigen-presenting cells (APCs), by downmodulating regulatory T cells (Tregs) and decreasing Myeloid-derived suppressor cells (MDSCs) which compose the so-called ‘immune permissive environment’ [45,46]. Moreover, cisplatin was also described to induce PD-L1 expression in vitro and in vivo [47,48,49,50,51], a co-inhibitory factor of the immune response which in turn augments the sensitivity of cancer cells to therapies using immune checkpoint inhibitors (ICIs) against PD-L1 [52,53]. In keeping with this, Fujita et al., demonstrated that miR-197-3p modulated cisplatin resistance by targeting the cyclin-dependent kinase CKS1B and, indirectly, the transcription factor STAT3, which ultimately leads to increased PD-L1 expression thus sensitizing PD-L1^high^ tumor cells to cisplatin [47]. Alternatively, an independent report showed that LncRNA MALAT1 induces chemoresistance of NSCLC cells through direct regulation of p120 catenin by competing with miR-197-3p [54].

Other miRNAs were found functionally involved in modulating cisplatin resistance though the exact molecular mechanisms were unclear [22,55,56,57,58].

### 2.4. miRNA Regulation of Platinum-Based Therapy Response

Cisplatin is the standard first-line treatment for advanced-stage NSCLC. It can be administered in combination with third-generation anticancer agents such as Gemcitabine, Docetaxel, Paclitaxel, or Vinorelbine, thus also called platinum-based doublet chemotherapy aka P-doublet [59]. As shown in Figure 1, a lower number of miRNAs was found involved in regulating responses to P-doublet in NSCLC, which is mainly ascribable to a lower number of research studies that analyzed miRNAs roles in P-doublet therapy. However, Lin et al. showed that miR-30-5p expression impairs resistance to cisplatin alone or in combination with pemetrexed [60] while it enhances paclitaxel sensitivity in an independent study [61]. Cai et al. found that miR-128-3p upregulation increased resistance to multiple drugs though used alone (cisplatin, gemcitabine, or paclitaxel; [62]), probably suggesting a similar effect when combined therapy would be otherwise used. Other miRNAs were also identified to regulate sensitivity to both cisplatin and paclitaxel (i.e., miR-186-5p [63,64]; miR-17-5p [22,65,66]; miR-34c-3p [67]) or cisplatin and docetaxel (miR-141-3p [68,69]; and miR-379-5p [70,71]).

### 2.5. miRNAs Which Modulate Response to EGFR Inhibitors

Mutations in EGFR are frequent in NSCLC patients (~10–20% of Caucasian, and up to 60% of South-East Asian patients) and cause the constitutive ligand-independent activation of EGFR receptor thus promoting cell growth and survival [72]. EGFR tyrosin kinase inhibitors (TKIs) are small molecules that bind the adenosine triphosphate (ATP) pocket of EGFR and inhibit its autophosphorylation and downstream signal transduction [73], including RAS–MAPK, PI3K–AKT, and JAK–STAT signaling. Initially, first- (gefitinib, erlotinib) and second-generation TKIs (afatinib, dacomitinib) have been developed and showed a better efficacy vs. platinum-based therapy alone in EGFR-mutated patients [74,75,76]. More recently, third-generation TKIs, such as Osimertinib, have shown a significant improvement in progression-free survival and overall survival [77], thus becoming the front-line therapy for patients with EGFR-mutated NSCLC [78]. Despite the initial response, resistance to EGFR TKIs inevitably occurs through both EGFR-target-dependent and independent mechanisms [79]. Some miRNAs were shown to modulate sensitivity to Gefitinib, Erlotinib, Afatinib, and Osimertinib with a high level of evidence (score ≥ 4 or 5) (Figure 1). In this context, transcriptome rewiring of TKIs resistant cells upon miRNA induction would activate alternative signaling pathways to bypass EGF inhibition [79]. For example, the rescue of miR-19a expression reverses gefitinib resistance in NSCLC by directly targeting 3′UTR of c-MET [80] which is one of the most altered pathways implicated in EGFR-TKIs inhibition [3,79]. Loss of PTEN was associated with both primary and acquired EGFR-TKIs resistance due to an increased PI3K signaling. Accordingly, miR-21 overexpression is associated with acquired resistance to gefitinib via inhibition of PTEN and PDCD4 and induction of PI3K/AKT [33,81]. Notably, increased expression of miR-21 was associated with afatinib resistance in both in vitro experimental models and in patients [82].

Other pathways were implicated in modulating sensitivity to EGFR-TKIs such as the regulation of NF1 which is a GTPase that negatively regulates the Ras/MEK/ERK pathway and is targeted by miR-641 regulation [83]. Yet, miR-326 impairs resistance to Gefitinib by direct inhibition of the type I interferon receptor IFNAR2 [84], which is relevant for JAK-STAT signaling activation [85] and is associated with the response to EGFR-TKIs [86]. Likewise, miR-762 is a potential downstream effector of the IL-6/STAT3 pathway and regulates gefitinib resistance via direct regulation of the target gene ABR [87]. Yet, loss of expression of miR-206 induces the over-activation of STAT3 which leads to Gefitinib resistance [88]. Furthermore, miR-206 have been reported to regulate response to gefinitib via other molecular mechanisms, including the direct regulation of the multidrug resistance protein ABCB1 [89] and by overcoming HGF activity (i.e., the ligand of Met) and promote MAPK and PI3K/AKT downstream pathways [90].

Gefinitib activity can be also modulated by the regulation of miR-146b-5p/nuclear factor kB (NF-kB) axis which impacts the NF-kB-related IL-6 and IL-8 production and ultimately enhances gefitinib-induced apoptosis [91]. Other miRNAs can influence Gefitinib response by regulating phenotypic traits of resistant cells. For example, Let-7 downregulation concomitantly increases in vitro self-renewal capability and Gefitinib resistance via direct regulation of MYC [92]. Furthermore, miR-17-5p increases tumor sphere formation and Gefitinib resistance by targeting CDKN1A (aka p21) [92,93]. This miRNA has also been associated with Erlotinib resistance although the molecular mechanisms have not been yet investigated [94]. Modulation of YAP1 gene expression by miR-7 is another mechanism of Gefitinib resistance via induction of EMT. Indeed, YAP1 was found to be targeted by miR-7 also through exosomal miR-7 transfer [95]. Similarly, miR-200c-3p inhibits downstream signaling pathways of EGFR and regulates both EMT and gefitinib resistance [96,97]. MiR-124-3p was found to be downregulated in gefitinib-resistant NSCLC patients, and its overexpression reversed EMT transformation and gefitinib resistance, at least in part, by the direct regulation of SNAI2 and STAT3 [98]. Contrariwise, miR-124-3p inhibition was associated with an increased sphere-forming efficiency and gefitinib resistance of EGFR-mutated cells through the unleashed expression of USP14 [99].

Other miRNAs were reported to regulate response to first-generation EGFR-TKIs through direct regulation of newly identified target genes including PELI3 by miR-365a-5p [100]; ATG5 by miR-153-3p [101]; TGFBR2 by miR-942-5p [100] and LHX6 by miR-214 [102]. Lastly, exosomal miR-184 was recently proposed as a biomarker resistance to third-generation TKI such as Osimertinib, which was reported to alter AKT phosphorylation and Osimertinib-induced cell death in cooperation with miR-22-3p [103,104]

Further studies are urgently needed to better understand the role of miRNAs in the regulation of response to third-generation TKIs, with a particular focus on atypical *EGFR* mutations that showed low response to EGFR-TKIs.

### 2.6. miRNAs Associated to Resistance to Radiotherapy

Radiotherapy is an important component of the multi-modality treatment for metastatic NSCLC, with ~50% of NSCLC patients undergoing radiotherapy either for a curative or palliative intent [105]. Radiotherapy kills tumor cells either by directly inducing DNA damage, which leads to cell death as well as increases anti-tumor immune response [106] or by indirectly altering tumor vasculature [107]. As said before, a large fraction of miRNAs associated with radiotherapy was downregulated in radioresistant samples and vice versa upregulated in radiosensitive ones, for example:(i) miR-218-5p overexpression restored the sensitivity of radiation-resistant cells through direct regulation of PRKDC, a member of the non-homologous end-joining pathway in the DNA double-strand break repair response [108]; (ii) rescue of miR-126-3p expression increased radiation-induced apoptosis by inactivation of the PI3K/AKT signaling [109]. MiR-449a, a p53-responsive miRNA, was found to regulate LDHA expression, which in turn inhibited glycolysis and increased radiotherapy sensitivity [110]; (iii) miR-375-3p and miR-513a-3p were respectively sponged by circ_0086720 and LINC00473, and their inhibition attenuated the knockdown-mediated radiosensitivity induced by these two ncRNAs [111,112]; (iv) re-expression of members of the let-7 family was functionally associated to increased radiosensitivity in NSCLC cells, although the molecular mechanisms were not fully investigated [102]; (v) miR-21-5p was found overexpressed in radioresistant samples while its inhibition was able to increase the sensitivity to radiotherapy through several molecular mechanisms including direct regulation of PTEN and PDCD4 genes, inactivation of the PI3K/AKT signaling or through modulation of HIF1-alpha dependent metabolism regulation [113,114,115,116,117]. Similarly, miR-25-3p was upregulated in radiation-resistant patients and was found to affect radiotherapy sensitivity by direct regulation of the newly identified target gene BTG2, a cell cycle modulator that acts as an effector of p53-induced cell cycle arrest [118].

### 2.7. Overview of the Experimental Strategies to Investigate the Functional Role of miRNA in NSCLC Therapy Response: Advantages and Limitations

The role of miRNA in intrinsic/acquired drug resistance in NSCLC has been mainly investigated using in vitro and in vivo experimental models such as tumor cell lines and mice xenografts and ex vivo samples such as blood and tumor tissues, which were used alone or in combination. Here below, we discuss the most frequently used experimental strategies, highlighting the pros and cons of such methodologies.

#### 2.7.1. Cell Lines

Tumors can be intrinsically resistant to therapy or, alternatively, can become resistant during the course of the treatment (acquired resistance). Overall, we found that a large fraction of studies were performed using in vitro model of acquired resistance which, in our opinion, could represent a potential bias as we later discuss. To get an in vitro model of acquired resistance, commercially available NSCLC cell lines are continuously exposed to increasing high doses of a drug to augment their resistance [119]. Drug response of a cell line before and after treatment is then measured by means of its half-maximal inhibitory concentration (IC_50_) which is the concentration of a drug that induces cell death of 50% of the bulk population. As an alternative, drug response metrics can be expressed as GR_50_ (drug-induced Growth Rate inhibition) which is less sensitive to growth rate differences influenced, for example, by the cell culture conditions or duration of an experiment [120]. We found that the A549 and NCI-H1299 cell lines were the most used for the generation of resistant variants to conventional chemotherapeutic agents and radiotherapy, while EGFR mutated cell lines, such as PC9 and NCI-H1975, were preferentially adopted in the case of EGFR TKIs treatment. To identify miRNAs associated with therapy resistance, quantitative real-time PCR (qPCR) or microarray analysis was preferred to map miRNA expression profiles between resistant and sensitive cell lines. One of the main advantages of the cell culture model of acquired resistance is isogenicity, which allows us to study miRNA function in cells with the same genetic background, thus reducing experimental variability. Moreover, the acquired resistance is stable and of high magnitude, which facilitates the propagation of persister cells (i.e., subpopulations of cells resistant to therapy) in cell culture, and it is generally associated with larger molecular changes to study [119]. Yet, these experimental approaches do not faithfully recapitulate neither the clinical setting where drugs are usually given in cycles at the same dose (pulsed treatment strategy) nor the intra-tumor heterogeneity observed in human cancer [121]. In addition, in many reports, the experimental details used to derive these models (e.g., the number of drug cycles, stability of the resistant phenotype, and timing of analysis of miRNA expression) were only partially described, thus affecting data reproducibility.

As an alternative to acquired resistance models, few other studies performed correlation analysis between drug sensitivity metrics of treatment-naive cells and miRNA expression profile to investigate miRNAs associated with intrinsic drug resistance. In this context, the use of primary cell cultures isolated directly from patient specimens (fine-needle aspirates [122], pleural effusion [123], or resection [124]) has been demonstrated as a reliable tool to predict therapy response [125]. A limitation of intrinsic drug resistance models can be the different genetic backgrounds among cell lines which could influence the final interpretation of the results. However, such potential limitation can be mitigated in silico by taking advantage of a large pharmacological screening (e.g., CTRPv2, GDSC1-2, and PRISM databases) coupled to public databases, such as Cancer Cell line Encyclopedia (CCLE) that contains the molecular profiling (methylation, mutation, miRNA and mRNA profiling, proteomics) of hundreds of cellular models [126]. In the last years, tumor samples were used also to establish 3D organoid cultures representing different tumor types, including lung cancers. In comparison to 2D in vitro cultures, organoids were shown to better retain essential histologic and molecular features of the parental tumors and to be highly concordant in terms of drug response with the tumors they were derived from [127]. This strongly suggests that the adoption of these 3D models would increase the resolution of experimental approaches to decipher miRNA activity in the context of therapy resistance.

Most of the miRNAs were found downregulated in persister tumor cells and resistant tumors. Therefore, it was not surprising for us to observe that miRNA overexpression was the most frequently used approach to investigate their role in drug response. Transient transfection of double-stranded RNA molecules that mimic the Dicer cleavage product of miRNA (mimics) or alternatively recombinant expression vectors carrying precursor or mature miRNA sequences are both very common strategies to overexpress miRNAs [128]. However, there are some potential pitfalls in such approaches, for example, they do not recapitulate the physiological maturation of a miRNA [9]. Likewise, overexpression of a miRNA can potentially saturate RNA-induced silencing complexes (RISCs) and displace other unrelated miRNAs with a dramatic impact on the modulation of the transcriptome in the engineered cells [129].

Contrariwise, miRNA inhibition can be used to knock down a specific miRNA by using for example: (i) antagomirs that are synthetic oligonucleotides designed to be complementary to specific miRNAs, thus preventing their binding to mRNA targets [130]; (ii) miRNA sponges that are constructed harboring multiple MREs which sequester endogenous miRNAs, thus preventing their regulation of mRNA targets [131]; (iii) CRISPR/Cas9-based editing approaches to knock-out miRNA genes [132]. Overall, transient transfection of oligonucleotides (i.e., antagomirs, sponges) have the limit to monitor only short-term effects and to be bypassed by compensatory molecular mechanisms ultimately masking phenotypes. It should also be kept into consideration that the expression of miRNA-targeted genes can be heterogenous (e.g., low/high expressed, not expressed) among tumor samples/cell lines which might ultimately influence the phenotypes observed upon modulation of candidate miRNAs.

Besides the identification of a functional role of miRNAs in drug response, some studies have also attempted to identify the exact molecular mechanisms. In supplementary Table S2, we listed genes that were both experimentally validated as direct targets of miRNAs and demonstrated, through rescue experiments, to be involved in miRNA-mediated regulation of drug resistance. It is worth noting that only a minor part of our reviewed studies (~27%) identified a miRNA-target gene axis that regulates drug resistance (Table S2). In these studies, an in-silico target prediction analysis (e.g., TargetScan, PicTar, miRanda, miRWalk) was initially performed, usually followed by gene expression profiling analysis upon miRNA expression modulation (overexpression/inhibition) as well as by experimental validation of the target genes (by cloning the 3′-UTR region of the gene of interest in a luciferase reported vector). However, this strategy did not consider miRNA binding sites located outside the 3′-UTR [133,134] and did not reflect physiological miRNA-to-MRE stoichiometries. Moreover, predicted MRE(s) can work efficiently in the luciferase-based assay but not in pathophysiological conditions where the loss of MREs could occur due to mutation, single nucleotide polymorphisms (SNPs), or proximal poly(A) termination [135].

Notably, some researchers performed also rescue experiments in which resistance was evaluated upon simultaneous miRNA inhibition and silencing of the target gene or, alternatively, miRNA overexpression and re-expression of the target genes [132]. Recently, target site blockers (TSBs) were used and shown to be valuable to study the effects of a miRNA on a single target. These are oligonucleotides that bind to specific MREs in 3′UTR of the target genes, thus preventing miRNA-target interactions in its endogenous cellular context. In comparison to miRNA inhibitors, TSBs did not interfere with miRNA activity thereby not altering the expression of other targets of the miRNAs [136].

#### 2.7.2. Animal Models

In vivo validation of the miRNA function in the context of the NSCLC therapy is generally performed by taking advantage of immunodeficient mice (e.g., NOD SCID). Cells are either stably or transiently transfected in vitro with miRNA inhibitors or mimics and injected in mice which are then treated with drugs (xenograft mouse model). Differences in primary tumor growth were then evaluated by using a caliper or, in the case of luciferase reporter constructs, by measuring bioluminescence signals. As an alternative, cell lines can be injected in nude mice which are then treated with the drug alone or in combination with miRNA mimics/inhibitors administered in vivo by intravenous, intraperitoneal, or intratumoral injection. However, a pitfall in this second approach is the impossibility to distinguish whether the biological effect of miRNA expression modulation is exerted exclusively in tumor cells or also in other cell types composing the tumor-microenvironment (TME).

Although xenograft models better mimic the tumor microenvironment than models used in in vitro studies, potential limitations arise from the use of cell-line-based xenografts (CDX) mouse models, such as (i) cell lines are continuously propagated in vitro, therefore, after several passages, they may lose resemblance with the original tumor from which they derived; (ii) xenografted cells usually form highly proliferating tumor lesions lacking the architecture of the original tumor and often characterized by hypoxic and/or necrotic regions due to variable perfusions [137]. Thus, the evaluation of drug efficacy can be affected by abnormal drug distribution or by the presence of actively replicating cells, especially in the case of cytotoxic drugs [138]. These limitations can be mitigated by the use of patient-derived xenograft (PDX) models, which were demonstrated to better retain tumor architecture likewise genetic and histological features, thus more resembling the original lesions despite several passages in vivo [139]. Furthermore, the subcutaneous injection did not faithfully recapitulate the complexity of the lung microenvironment and the evolution of the metastatic process [140]. For example, the dissemination of cancer cells in organs characterized by a soft environment could alter metastatic growth and therapy response [141]. To overcome at least in part such limitations, Fujita et al., analyzed the role of miR-197 by taking advantage of an orthotopic xenograft model in which they directly injected miRNA-stable transfected cell lines in the lung of nude mice [47].

The lack of a competent immune system in immunodeficient mice is a major limitation when studying the efficacy and/or mechanisms of resistance to immunotherapy [142]. To solve this problem, it is possible to take advantage of syngeneic mouse models in which murine cells were injected into immunocompetent mice. However, some miRNA-mRNA interactions could not be conserved across mouse to human due to differences in miRNAs sequences or in the 3′UTR region of the miRNA target [143]. As an alternative to these experimental strategies, humanized mouse models have recently been developed and could be used to study the role of miRNA in resistance to therapeutic agents that affect immune response (e.g., ICIs) [144].

#### 2.7.3. Human Cancer Tissue

The role of miRNA in determining drug-resistant phenotypes can be further validated using ex vivo models derived from patient biopsies. The remarkable stability of miRNAs even in harsh conditions such as formalin fixed paraffin-embedded (FFPE) tissue or body fluids (e.g., plasma, serum, urine) definitely contributed to the success of miRNA expression profile analysis in tumor biopsies [145,146]. As we previously discussed, in vitro models based on tumor cell lines are useful to explore the biological role of a miRNA but they have an intrinsic limitation i.e., they are a homogeneous population of cells of clonal origin. Contrariwise, ex vivo models from human tumor biopsies can better represent the community of tumor cells with heterogenous genetic and epigenetic background intertwined with other cell types (e.g., immune cells, fibroblast, endothelial cells, and normal epithelial cells) which compose the TME. Therefore, when investigating miRNA regulation in the context of drug response, ex vivo models are pivotal for a better understanding of the functional role of miRNAs in human tumor samples.

**Figure 2:**
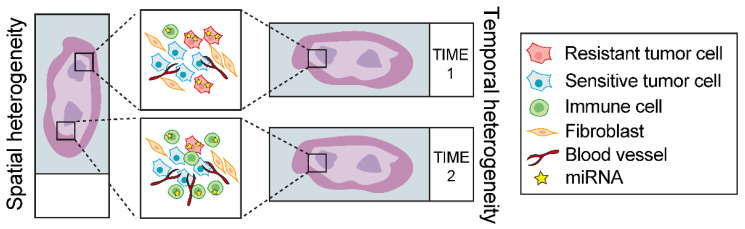
The problem of tumor heterogeneity in the study of miRNAs associated to therapy response. Schematic model of how spatial and/or temporal heterogeneity can affect the analysis of the association between miRNA expression and therapy response.

However, some limitations still apply for example in the bulk tumor miRNA expression profile analysis, which does not provide any clues about miRNA expression profile in specific cell types but rather gives an average expression quantification between tumor cells and TME. Notably, intratumoral biological and genetic heterogeneity bolsters the acquisition of drug resistance because of the presence of a treatment-resistant subpopulation of cells that can outgrow during the course of the treatment. Alternatively, some cells have the ability to survive initial treatment (i.e., drug-tolerant ‘persister’ cells) and acquire de-novo genetic and/or epigenetics alterations that give therapeutic resistance [147]. Such heterogeneity, which is functional to therapy resistance can be either spatial, where there is an uneven distribution of tumor subpopulation across different regions of the tumor, or temporal, which refers to a molecular variation of the tumor over time (Figure 2) [148,149,150]. Yet, differences in tumor purity (i.e., percentage of tumor cells in the tissue) may also puzzle the association of miRNAs expression with therapy response or with target gene expression thus leading to suboptimal assessments of correlation scores [151]. In addition, TME cellular components are known to reprogram the molecular profile and phenotypes of cancer cells [152,153,154,155], thus a different composition of TME and relative abundance in the cohort of samples analyzed can potentially hide a specific miRNA-related phenotype (Figure 2).

Lastly, ex vivo models may suffer from an additional potential limitation which relies on the use of samples collected mainly from primary lesions instead of metastases. Of course, it is more common during surgery to collect biospecimen from primary lesions than from metastases; indeed, surgery is not an option for advanced metastatic disease [156]. However, the majority of approved cancer drugs do not have a long-term impact in the setting of metastatic disease, which suggests that a better characterization of underlying molecular mechanisms and evolutionary patterns in lung cancer metastases is paramount [157]. For example, EGFR-mutated lung metastatic cells have been recently shown to be highly dependent on the S100A9-ALDH1A1-RA axis for growth in the brain but not in other distant sites such as the bone or the lung [158]. Similarly, lung metastases exhibited an elevated mTORC1 growth signaling due to a higher pyruvate uptake and serine biosynthesis in comparison to primary breast cancer cells from which they originated [159]. For these reasons, we think that a comprehensive analysis of drug tolerance mechanisms should be performed in both primary and metastatic samples.

## 3. Conclusions and Discussion

Here, we made an update on recent research about the functional role of miRNAs in the regulation of resistance to drugs that are currently approved in NSCLC. Overall, miRNAs were more frequently downregulated in drug-resistant than in sensitive samples, and virtually all of the known molecular mechanisms that drive drug resistance in NSCLC cells were found targeted by miRNAs.

The number of therapeutic agents approved by the FDA for NSCLC treatment has continuously increased in the last years and, for some drugs, we scored only few or no miRNAs reported to have a functional role. ICIs (e.g., Nivolumab, Pembrolizumab, Atezolizumab, and Durvalumab) have recently become the standard of care for patients with advanced NSCLC disease [160] thus opening a new era for the clinical management of metastatic patients. Change in the expression of selected miRNAs has been reported when plasma and serum of patients treated with either anti-PD1 or anti-PD-L1 antibodies have been investigated [161,162,163,164,165,166]. These preclinical studies highlighted the potential role of miRNAs as biomarkers predictive of response to ICIs. It is also conceivable that future functional studies will demonstrate the role of miRNAs in the modulation of immunotherapy response. Moreover, a number of clinical studies are now evaluating the effect of combination of a conventional chemotherapy with ICIs in neo-adjuvant and adjuvant settings, with very promising preliminary results [167]. In keeping with this, it would be interesting to study how the miRNAs, reported to be functionally involved in chemoresistance, can impact also the response to ICIs when used alone or in combination with chemotherapy.

In conclusion, we note that current limitations in the research field of miRNAs and drug resistance may include: (i) weaknesses of “classical” experimental models such as commercially available 2D in vitro cultures and xenograft mouse models; (ii) intrinsic limitation of methodologies to study miRNA function; (iii) tumor cell heterogeneity and interaction with TME which make the identification of cell-type specific mechanisms involved in drug tolerance complicated. More advanced technologies and experimental models to fully recapitulate the tumor architecture, as well as the molecular and biological heterogeneity of lung cancer, are therefore recommended.

A limit of our review could be related to a lack of investigation about the specificity of the miRNAs discussed in terms of eventual similar functions/mechanisms of action in other types of cancers.

## Figures and Tables

**Table 1:** List of miRNAs implicated in common resistance mechanisms to drugs (“Drug”) as well as modulating shared pathways/signalling cascades (“Common Pathway/signalling”). MicroRNA ID (“miRNA”) likewise pubmed reference ID (“PMID”) is also reported.

Common Pathway/Signalling	Drug	miRNA	Reference (PMID)
Autophagy	Cisplatin	miR-1-3p	29851226
	Cisplatin	miR-425-3p	31632022
	Gefitinib	miR-153-3p	30964170
Cell cycle and DNA repair	Cisplatin	miR-92a-3p	26482648
	Cisplatin	miR-4458	32808744
	Radiotherapy	miR-218-5p	33759399
	Cisplatin	miR-17-5p	26482648
	Radiotherapy	miR-25-3p	25576360
Drug transport	Cisplatin	miR-369-3p	28511796
	Cisplatin	miR-495-3p	24038379
	Gefitinib	miR-206	31121484
EMT and/or Stem-like properties	Cisplatin	miR-128-3p	28627514
	Cisplatin	miR-181b-5p	26620926, 30470250
	Gefitinib	miR-200c-3p	27930974
	Gefitinib	miR-124-3p	29702194, 27924500
	Gefitinib	Let-7	31233201
	Gefitinib	miR-17-5p	31233201
MET signalling	Gefitinib	miR-19a	28592790
	Gefitinib	miR-206	33955799
Metabolism	Radiotherapy	MiR-449a	28800787
	Radiotherapy	miR-21-5p	27035555
PI3K/AKT signalling	Cisplatin	miR-29c-3p	29789623
	Cisplatin	miR-126-5p	34055989
	Cisplatin	miR-539-5p	30119173
	Cisplatin	miR-21-5p	22237007, 22956424, 28686971
	Gefitinib	miR-21-5p	25058005
	Gefitinib	miR-206	33955799
	Osimertinib	miR-184	35461372
	Osimertinib	miR-22-3p	35461372
	Radiotherapy	miR-126-3p	20728239
	Gefitinib	miR-200c-3p	27930974
	Radiotherapy	miR-21-5p	32020207, 24804226, 22956424
RAS signalling	Cisplatin	miR-103a-3p	32104235
	Erlotinib	miR-641	29493886
STAT signalling	Gefitinib	miR-326	35081872
	Gefitinib	miR-762	25597412
	Gefitinib	miR-206	31507089
	Cisplatin	miR-197-3p	25597412

## Supplementary Materials

The following supporting information can be downloaded at: https://www.mdpi.com/article/10.3390/cancers14235731/s1, Supplementary Table S1. List of the drugs currently used in clinics to treat NSCLC patients according to the NCCN guidelines (Version 3.2022). Supplementary Table S2. List of miRNAs associated to therapy response in NSCLC. Supplementary Table S3. List of miRNAs as shown in Figure 1B.

## References

[1] Miller K.D., Nogueira L., Devasia T., Mariotto A.B., Yabroff K.R., Jemal A., Kramer J., Siegel R.L. (2022). Cancer Treatment and Survivorship Statistics, 2022. CA Cancer J. Clin..

[2] Chen Z., Fillmore C.M., Hammerman P.S., Kim C.F., Wong K.-K. (2014). Non-Small-Cell Lung Cancers: A Heterogeneous Set of Diseases. Nat. Rev. Cancer.

[3] Lim Z.-F., Ma P.C. (2019). Emerging Insights of Tumor Heterogeneity and Drug Resistance Mechanisms in Lung Cancer Targeted Therapy. J. Hematol. Oncol..

[4] Yanaihara N., Caplen N., Bowman E., Seike M., Kumamoto K., Yi M., Stephens R.M., Okamoto A., Yokota J., Tanaka T. (2006). Unique MicroRNA Molecular Profiles in Lung Cancer Diagnosis and Prognosis. Cancer Cell.

[5] Cancer Genome Atlas Research N. (2014). Comprehensive Molecular Profiling of Lung Adenocarcinoma. Nature.

[6] Dama E., Melocchi V., Colangelo T., Cuttano R., Bianchi F. (2019). Deciphering the Molecular Profile of Lung Cancer: New Strategies for the Early Detection and Prognostic Stratification. J. Clin. Med..

[7] Si W., Shen J., Zheng H., Fan W. (2019). The Role and Mechanisms of Action of MicroRNAs in Cancer Drug Resistance. Clin. Epigenet..

[8] Treiber T., Treiber N., Meister G. (2019). Regulation of MicroRNA Biogenesis and Its Crosstalk with Other Cellular Pathways. Nat. Rev. Mol. Cell Biol..

[9] Dragomir M.P., Knutsen E., Calin G.A. (2022). Classical and Noncanonical Functions of MiRNAs in Cancers. Trends Genet..

[10] Chevillet J.R., Kang Q., Ruf I.K., Briggs H.A., Vojtech L.N., Hughes S.M., Cheng H.H., Arroyo J.D., Meredith E.K., Gallichotte E.N. (2014). Quantitative and Stoichiometric Analysis of the MicroRNA Content of Exosomes. Proc. Natl. Acad. Sci. USA.

[11] Turchinovich A., Weiz L., Langheinz A., Burwinkel B. (2011). Characterization of Extracellular Circulating MicroRNA. Nucleic Acids Res..

[12] Arroyo J.D., Chevillet J.R., Kroh E.M., Ruf I.K., Pritchard C.C., Gibson D.F., Mitchell P.S., Bennett C.F., Pogosova-Agadjanyan E.L., Stirewalt D.L. (2011). Argonaute2 Complexes Carry a Population of Circulating MicroRNAs Independent of Vesicles in Human Plasma. Proc. Natl. Acad. Sci. USA.

[13] Vickers K.C., Palmisano B.T., Shoucri B.M., Shamburek R.D., Remaley A.T. (2011). MicroRNAs Are Transported in Plasma and Delivered to Recipient Cells by High-Density Lipoproteins. Nat. Cell Biol..

[14] Pardini B., Calin G.A. (2019). MicroRNAs and Long Non-Coding RNAs and Their Hormone-Like Activities in Cancer. Cancers.

[15] Kelland L. (2007). The Resurgence of Platinum-Based Cancer Chemotherapy. Nat. Rev. Cancer.

[16] Kumar M.S., Lu J., Mercer K.L., Golub T.R., Jacks T. (2007). Impaired MicroRNA Processing Enhances Cellular Transformation and Tumorigenesis. Nat. Genet..

[17] Yuan M., Huang L.-L., Chen J.-H., Wu J., Xu Q. (2019). The Emerging Treatment Landscape of Targeted Therapy in Non-Small-Cell Lung Cancer. Signal Transduct. Target. Ther..

[18] Rottenberg S., Disler C., Perego P. (2021). The Rediscovery of Platinum-Based Cancer Therapy. Nat. Rev. Cancer.

[19] Huang D., Savage S.R., Calinawan A.P., Lin C., Zhang B., Wang P., Starr T.K., Birrer M.J., Paulovich A.G. (2021). A Highly Annotated Database of Genes Associated with Platinum Resistance in Cancer. Oncogene.

[20] Hao G.-J., Ding Y.-H., Wen H., Li X.-F., Zhang W., Su H.-Y., Liu D.-M., Xie N.-L. (2017). Attenuation of Deregulated MiR-369-3p Expression Sensitizes Non-Small Cell Lung Cancer Cells to Cisplatin via Modulation of the Nucleotide Sugar Transporter SLC35F5. Biochem. Biophys. Res. Commun..

[21] Song L., Li Y., Li W., Wu S., Li Z. (2014). MiR-495 Enhances the Sensitivity of Non-Small Cell Lung Cancer Cells to Platinum by Modulation of Copper-Transporting P-Type Adenosine Triphosphatase A (ATP7A). J. Cell. Biochem..

[22] Zhao J., Fu W., Liao H., Dai L., Jiang Z., Pan Y., Huang H., Mo Y., Li S., Yang G. (2015). The Regulatory and Predictive Functions of MiR-17 and MiR-92 Families on Cisplatin Resistance of Non-Small Cell Lung Cancer. BMC Cancer.

[23] Huang Q., Xing S., Peng A., Yu Z. (2020). NORAD Accelerates Chemo-Resistance of Non-Small-Cell Lung Cancer via Targeting at MiR-129-1-3p/SOX4 Axis. Biosci. Rep..

[24] Pang J., Ye L., Zhao D., Zhao D., Chen Q. (2020). Circular RNA PRMT5 Confers Cisplatin-Resistance via MiR-4458/REV3L Axis in Non-Small-Cell Lung Cancer. Cell Biol. Int..

[25] Zhu H., Yang J., Yang S. (2020). MicroRNA-103a-3p Potentiates Chemoresistance to Cisplatin in Non-Small Cell Lung Carcinoma by Targeting Neurofibromatosis 1. Exp. Ther. Med..

[26] Wang H., Huang H., Wang L., Liu Y., Wang M., Zhao S., Lu G., Kang X. (2021). Cancer-Associated Fibroblasts Secreted MiR-103a-3p Suppresses Apoptosis and Promotes Cisplatin Resistance in Non-Small Cell Lung Cancer. Aging.

[27] Sun D.-M., Tang B.-F., Li Z.-X., Guo H.-B., Cheng J.-L., Song P.-P., Zhao X. (2018). MiR-29c Reduces the Cisplatin Resistance of Non-Small Cell Lung Cancer Cells by Negatively Regulating the PI3K/Akt Pathway. Sci. Rep..

[28] Liu B., Wang R., Liu H. (2021). Mir-126-5p Promotes Cisplatin Sensitivity of Non-Small-Cell Lung Cancer by Inhibiting ADAM9. BioMed Res. Int..

[29] Deng H., Qianqian G., Ting J., Aimin Y. (2018). MiR-539 Enhances Chemosensitivity to Cisplatin in Non-Small Cell Lung Cancer by Targeting DCLK1. Biomed. Pharmacother..

[30] Ge P., Cao L., Zheng M., Yao Y., Wang W., Chen X. (2021). LncRNA SNHG1 Contributes to the Cisplatin Resistance and Progression of NSCLC via MiR-330-5p/DCLK1 Axis. Exp. Mol. Pathol..

[31] Wei J., Gao W., Zhu C.-J., Liu Y.-Q., Mei Z., Cheng T., Shu Y.-Q. (2011). Identification of Plasma MicroRNA-21 as a Biomarker for Early Detection and Chemosensitivity of Non-Small Cell Lung Cancer. Chin. J. Cancer.

[32] Gao W., Lu X., Liu L., Xu J., Feng D., Shu Y. (2012). MiRNA-21: A Biomarker Predictive for Platinum-Based Adjuvant Chemotherapy Response in Patients with Non-Small Cell Lung Cancer. Cancer Biol. Ther..

[33] Liu Z.-L., Wang H., Liu J., Wang Z.-X. (2013). MicroRNA-21 (MiR-21) Expression Promotes Growth, Metastasis, and Chemo- or Radioresistance in Non-Small Cell Lung Cancer Cells by Targeting PTEN. Mol. Cell. Biochem..

[34] Xu L., Huang Y., Chen D., He J., Zhu W., Zhang Y., Liu X. (2014). Downregulation of MiR-21 Increases Cisplatin Sensitivity of Non-Small-Cell Lung Cancer. Cancer Genet..

[35] Yang Z., Fang S., Di Y., Ying W., Tan Y., Gu W. (2015). Modulation of NF-ΚB/MiR-21/PTEN Pathway Sensitizes Non-Small Cell Lung Cancer to Cisplatin. PLoS ONE.

[36] Cao L., Chen J., Ou B., Liu C., Zou Y., Chen Q. (2017). GAS5 Knockdown Reduces the Chemo-Sensitivity of Non-Small Cell Lung Cancer (NSCLC) Cell to Cisplatin (DDP) through Regulating MiR-21/PTEN Axis. Biomed. Pharmacother..

[37] Shi L., Middleton J., Jeon Y.-J., Magee P., Veneziano D., Laganà A., Leong H.-S., Sahoo S., Fassan M., Booton R. (2018). KRAS Induces Lung Tumorigenesis through MicroRNAs Modulation. Cell Death Dis..

[38] Li C., Fan K., Qu Y., Zhai W., Huang A., Sun X., Xing S. (2020). Deregulation of UCA1 Expression May Be Involved in the Development of Chemoresistance to Cisplatin in the Treatment of Non-Small-Cell Lung Cancer via Regulating the Signaling Pathway of MicroRNA-495/NRF2. J. Cell. Physiol..

[39] Shibue T., Weinberg R.A. (2017). EMT, CSCs, and Drug Resistance: The Mechanistic Link and Clinical Implications. Nat. Rev. Clin. Oncol..

[40] Cai J., Guan H., Fang L., Yang Y., Zhu X., Yuan J., Wu J., Li M. (2013). MicroRNA-374a Activates Wnt/Beta-Catenin Signaling to Promote Breast Cancer Metastasis. J. Clin. Investig..

[41] Wang X., Chen X., Meng Q., Jing H., Lu H., Yang Y., Cai L., Zhao Y. (2015). MiR-181b Regulates Cisplatin Chemosensitivity and Metastasis by Targeting TGFβR1/Smad Signaling Pathway in NSCLC. Sci. Rep..

[42] Wang X., Meng Q., Qiao W., Ma R., Ju W., Hu J., Lu H., Cui J., Jin Z., Zhao Y. (2018). MiR-181b/Notch2 Overcome Chemoresistance by Regulating Cancer Stem Cell-like Properties in NSCLC. Stem Cell Res. Ther..

[43] Hua L., Zhu G., Wei J. (2018). MicroRNA-1 Overexpression Increases Chemosensitivity of Non-Small Cell Lung Cancer Cells by Inhibiting Autophagy Related 3-Mediated Autophagy. Cell Biol. Int..

[44] Ma Y., Yuwen D., Chen J., Zheng B., Gao J., Fan M., Xue W., Wang Y., Li W., Shu Y. (2019). Exosomal Transfer Of Cisplatin-Induced MiR-425-3p Confers Cisplatin Resistance In NSCLC Through Activating Autophagy. Int. J. Nanomed..

[45] Rébé C., Demontoux L., Pilot T., Ghiringhelli F. (2019). Platinum Derivatives Effects on Anticancer Immune Response. Biomolecules.

[46] Hato S.V., Khong A., de Vries I.J.M., Lesterhuis W.J. (2014). Molecular Pathways: The Immunogenic Effects of Platinum-Based Chemotherapeutics. Clin. Cancer Res..

[47] Fujita Y., Yagishita S., Hagiwara K., Yoshioka Y., Kosaka N., Takeshita F., Fujiwara T., Tsuta K., Nokihara H., Tamura T. (2015). The Clinical Relevance of the MiR-197/CKS1B/STAT3-Mediated PD-L1 Network in Chemoresistant Non-Small-Cell Lung Cancer. Mol. Ther..

[48] Parra E.R., Villalobos P., Behrens C., Jiang M., Pataer A., Swisher S.G., William W.N., Zhang J., Lee J., Cascone T. (2018). Effect of Neoadjuvant Chemotherapy on the Immune Microenvironment in Non–Small Cell Lung Carcinomas as Determined by Multiplex Immunofluorescence and Image Analysis Approaches. J. Immunother. Cancer.

[49] Zhang P., Ma Y., Lv C., Huang M., Li M., Dong B., Liu X., An G., Zhang W., Zhang J. (2016). Upregulation of Programmed Cell Death Ligand 1 Promotes Resistance Response in Non-small-cell Lung Cancer Patients Treated with Neo-adjuvant Chemotherapy. Cancer Sci..

[50] Shin J., Chung J.-H., Kim S.H., Lee K.S., Suh K.J., Lee J.Y., Kim J.-W., Lee J.-O., Kim J.-W., Kim Y.-J. (2019). Effect of Platinum-Based Chemotherapy on PD-L1 Expression on Tumor Cells in Non-Small Cell Lung Cancer. Cancer Res. Treat..

[51] Guo L., Song P., Xue X., Guo C., Han L., Fang Q., Ying J., Gao S., Li W. (2019). Variation of Programmed Death Ligand 1 Expression After Platinum-Based Neoadjuvant Chemotherapy in Lung Cancer. J. Immunother..

[52] Fournel L., Wu Z., Stadler N., Damotte D., Lococo F., Boulle G., Ségal-Bendirdjian E., Bobbio A., Icard P., Trédaniel J. (2019). Cisplatin Increases PD-L1 Expression and Optimizes Immune Check-Point Blockade in Non-Small Cell Lung Cancer. Cancer Lett..

[53] Wakita D., Iwai T., Harada S., Suzuki M., Yamamoto K., Sugimoto M. (2019). Cisplatin Augments Antitumor T-Cell Responses Leading to a Potent Therapeutic Effect in Combination With PD-L1 Blockade. Anticancer Res..

[54] Yang T., Li H., Chen T., Ren H., Shi P., Chen M. (2019). LncRNA MALAT1 Depressed Chemo-Sensitivity of NSCLC Cells through Directly Functioning on MiR-197-3p/P120 Catenin Axis. Mol. Cells.

[55] Yin J., Zhao J., Hu W., Yang G., Yu H., Wang R., Wang L., Zhang G., Fu W., Dai L. (2017). Disturbance of the Let-7/LIN28 Double-Negative Feedback Loop Is Associated with Radio- and Chemo-Resistance in Non-Small Cell Lung Cancer. PLoS ONE.

[56] Zhao X., Wang J., Zhu R., Zhang J., Zhang Y. (2021). DLX6-AS1 Activated by H3K4me1 Enhanced Secondary Cisplatin Resistance of Lung Squamous Cell Carcinoma through Modulating MiR-181a-5p/MiR-382-5p/CELF1 Axis. Sci. Rep..

[57] Wang C., Wang S., Ma F., Zhang W. (2018). MiRNA-328 Overexpression Confers Cisplatin Resistance in Non-small Cell Lung Cancer via Targeting of PTEN. Mol. Med. Rep..

[58] Jiang Z., Yin J., Fu W., Mo Y., Pan Y., Dai L., Huang H., Li S., Zhao J. (2014). MiRNA 17 Family Regulates Cisplatin-Resistant and Metastasis by Targeting TGFbetaR2 in NSCLC. PLoS ONE.

[59] Hellmann M.D., Li B.T., Chaft J.E., Kris M.G. (2016). Chemotherapy Remains an Essential Element of Personalized Care for Persons with Lung Cancers. Ann. Oncol..

[60] Lin X., Lai X., Feng W., Yu X., Gu Q., Zheng X. (2021). MiR-30a Sensitized Lung Cancer against Neoadjuvant Chemotherapy by Depressing Autophagy. Jpn. J. Clin. Oncol..

[61] Xu X., Jin S., Ma Y., Fan Z., Yan Z., Li W., Song Q., You W., Lyu Z., Song Y. (2017). MiR-30a-5p Enhances Paclitaxel Sensitivity in Non-Small Cell Lung Cancer through Targeting BCL-2 Expression. J. Mol. Med..

[62] Cai J., Fang L., Huang Y., Li R., Xu X., Hu Z., Zhang L., Yang Y., Zhu X., Zhang H. (2017). Simultaneous Overactivation of Wnt/β-Catenin and TGFβ Signalling by MiR-128-3p Confers Chemoresistance-Associated Metastasis in NSCLC. Nat. Commun..

[63] Liu X., Zhou X., Chen Y., Huang Y., He J., Luo H. (2020). MiR-186-5p Targeting SIX1 Inhibits Cisplatin Resistance in Non-Small-Cell Lung Cancer Cells (NSCLCs). Neoplasma.

[64] Ye J., Zhang Z., Sun L., Fang Y., Xu X., Zhou G. (2016). MiR-186 Regulates Chemo-Sensitivity to Paclitaxel via Targeting MAPT in Non-Small Cell Lung Cancer (NSCLC). Mol. Biosyst..

[65] Chatterjee A., Chattopadhyay D., Chakrabarti G. (2014). MiR-17-5p Downregulation Contributes to Paclitaxel Resistance of Lung Cancer Cells through Altering Beclin1 Expression. PLoS ONE.

[66] Chatterjee A., Chattopadhyay D., Chakrabarti G. (2015). MiR-16 Targets Bcl-2 in Paclitaxel-Resistant Lung Cancer Cells and Overexpression of MiR-16 along with MiR-17 Causes Unprecedented Sensitivity by Simultaneously Modulating Autophagy and Apoptosis. Cell. Signal..

[67] Yang L.-Z., Lei C.-C., Zhao Y.-P., Sun H.-W., Yu Q.-H., Yang E.-J., Zhan X. (2020). MicroRNA-34c-3p Target Inhibiting NOTCH1 Suppresses Chemosensitivity and Metastasis of Non-Small Cell Lung Cancer. J. Int. Med. Res..

[68] Fu W.-F., Chen W.-B., Dai L., Yang G.-P., Jiang Z.-Y., Pan L., Zhao J., Chen G. (2016). Inhibition of MiR-141 Reverses Cisplatin Resistance in Non-Small Cell Lung Cancer Cells via Upregulation of Programmed Cell Death Protein 4. Eur. Rev. Med. Pharmacol. Sci.

[69] Wang D., Ma J., Ji X., Xu F., Wei Y. (2017). MiR-141 Regulation of EIF4E Expression Affects Docetaxel Chemoresistance of Non-Small Cell Lung Cancer. Oncol. Rep..

[70] Hao G.-J., Hao H.-J., Ding Y.-H., Wen H., Li X.-F., Wang Q.-R., Zhang B.-B. (2017). Suppression of EIF4G2 by MiR-379 Potentiates the Cisplatin Chemosensitivity in Nonsmall Cell Lung Cancer Cells. FEBS Lett..

[71] Zheng S., Wang C., Yan H., Du Y. (2021). Blocking Hsa_circ_0074027 Suppressed Non-Small Cell Lung Cancer Chemoresistance via the MiR-379-5p/IGF1 Axis. Bioengineered.

[72] Hsu W.-H., Yang J.C.-H., Mok T.S., Loong H.H. (2018). Overview of Current Systemic Management of EGFR-Mutant NSCLC. Ann. Oncol..

[73] Morin M.J. (2000). From Oncogene to Drug: Development of Small Molecule Tyrosine Kinase Inhibitors as Anti-Tumor and Anti-Angiogenic Agents. Oncogene.

[74] Sequist L.V., Yang J.C.-H., Yamamoto N., O’Byrne K., Hirsh V., Mok T., Geater S.L., Orlov S., Tsai C.-M., Boyer M. (2013). Phase III Study of Afatinib or Cisplatin plus Pemetrexed in Patients with Metastatic Lung Adenocarcinoma with EGFR Mutations. J. Clin. Oncol..

[75] Zhou C., Wu Y.-L., Chen G., Feng J., Liu X.-Q., Wang C., Zhang S., Wang J., Zhou S., Ren S. (2011). Erlotinib versus Chemotherapy as First-Line Treatment for Patients with Advanced EGFR Mutation-Positive Non-Small-Cell Lung Cancer (OPTIMAL, CTONG-0802): A Multicentre, Open-Label, Randomised, Phase 3 Study. Lancet Oncol..

[76] Mok T.S., Wu Y.-L., Thongprasert S., Yang C.-H., Chu D.-T., Saijo N., Sunpaweravong P., Han B., Margono B., Ichinose Y. (2009). Gefitinib or Carboplatin-Paclitaxel in Pulmonary Adenocarcinoma. N. Engl. J. Med..

[77] Soria J.-C., Ohe Y., Vansteenkiste J., Reungwetwattana T., Chewaskulyong B., Lee K.H., Dechaphunkul A., Imamura F., Nogami N., Kurata T. (2018). Osimertinib in Untreated EGFR-Mutated Advanced Non-Small-Cell Lung Cancer. N. Engl. J. Med..

[78] Cooper A.J., Sequist L.V., Lin J.J. (2022). Third-Generation EGFR and ALK Inhibitors: Mechanisms of Resistance and Management. Nat. Rev. Clin. Oncol..

[79] Passaro A., Jänne P.A., Mok T., Peters S. (2021). Overcoming Therapy Resistance in EGFR-Mutant Lung Cancer. Nat. Cancer.

[80] Cao X., Lai S., Hu F., Li G., Wang G., Luo X., Fu X., Hu J. (2017). MiR-19a Contributes to Gefitinib Resistance and Epithelial Mesenchymal Transition in Non-Small Cell Lung Cancer Cells by Targeting c-Met. Sci. Rep..

[81] Shen H., Zhu F., Liu J., Xu T., Pei D., Wang R., Qian Y., Li Q., Wang L., Shi Z. (2014). Alteration in Mir-21/PTEN Expression Modulates Gefitinib Resistance in Non-Small Cell Lung Cancer. PLoS ONE.

[82] Leonetti A., Capula M., Minari R., Mazzaschi G., Gregori A., El Hassouni B., Papini F., Bordi P., Verzè M., Avan A. (2021). Dynamic Evaluation of Circulating MiRNA Profile in EGFR-Mutated NSCLC Patients Treated with EGFR-TKIs. Cells.

[83] Chen J., Cui J., Guo X.-T., Cao X., Li Q. (2018). Increased Expression of MiR-641 Contributes to Erlotinib Resistance in Non-Small-Cell Lung Cancer Cells by Targeting NF1. Cancer Med..

[84] Zheng Y., Guo Z., Li Y. (2022). Long Non-Coding RNA Prostate Cancer-Associated Transcript 6 Inhibited Gefitinib Sensitivity of Non-Small Cell Lung Cancer by Serving as a Competing Endogenous RNA of MiR-326 to up-Regulate Interferon-Alpha Receptor 2. Bioengineered.

[85] Shemesh M., Lochte S., Piehler J., Schreiber G. (2021). IFNAR1 and IFNAR2 Play Distinct Roles in Initiating Type I Interferon–Induced JAK-STAT Signaling and Activating STATs. Sci. Signal..

[86] Gong K., Guo G., Panchani N., Bender M.E., Gerber D.E., Minna J.D., Fattah F., Gao B., Peyton M., Kernstine K. (2020). EGFR Inhibition Triggers an Adaptive Response by Co-Opting Antiviral Signaling Pathways in Lung Cancer. Nat. Cancer.

[87] Ge P., Cao L., Chen X., Jing R., Yue W. (2019). MiR-762 Activation Confers Acquired Resistance to Gefitinib in Non-Small Cell Lung Cancer. BMC Cancer.

[88] Yang Y., Wang W., Chang H., Han Z., Yu X., Zhang T. (2019). Reciprocal Regulation of MiR-206 and IL-6/STAT3 Pathway Mediates IL6-Induced Gefitinib Resistance in EGFR-Mutant Lung Cancer Cells. J. Cell. Mol. Med..

[89] Wu K., Li J., Qi Y., Zhang C., Zhu D., Liu D., Zhao S. (2019). SNHG14 Confers Gefitinib Resistance in Non-Small Cell Lung Cancer by up-Regulating ABCB1 via Sponging MiR-206-3p. Biomed. Pharmacother..

[90] Jiao D., Jiang C., Zhu L., Zheng J., Liu X., Liu X., Chen J., Tang X., Chen Q. (2021). MiR-1/133a and MiR-206/133b Clusters Overcome HGF Induced Gefitinib Resistance in Non-Small Cell Lung Cancers with EGFR Sensitive Mutations. J. Drug Target..

[91] Liu Y.-N., Tsai M.-F., Wu S.-G., Chang T.-H., Tsai T.-H., Gow C.-H., Wang H.-Y., Shih J.-Y. (2020). MiR-146b-5p Enhances the Sensitivity of NSCLC to EGFR Tyrosine Kinase Inhibitors by Regulating the IRAK1/NF-ΚB Pathway. Mol. Ther. Nucleic Acids.

[92] Yin J., Hu W., Pan L., Fu W., Dai L., Jiang Z., Zhang F., Zhao J. (2019). Let-7 and MiR-17 Promote Self-renewal and Drive Gefitinib Resistance in Non-small Cell Lung Cancer. Oncol. Rep..

[93] Gong J., He L., Ma J., Zhang J., Wang L., Wang J. (2017). The Relationship between MiR-17-5p, MiR-92a, and Let-7b Expression with Non-Small Cell Lung Cancer Targeted Drug Resistance. J. BUON.

[94] Zhang W., Lin J., Wang P., Sun J. (2017). MiR-17-5p down-Regulation Contributes to Erlotinib Resistance in Non-Small Cell Lung Cancer Cells. J. Drug Target..

[95] Chen R., Qian Z., Xu X., Zhang C., Niu Y., Wang Z., Sun J., Zhang X., Yu Y. (2021). Exosomes-Transmitted MiR-7 Reverses Gefitinib Resistance by Targeting YAP in Non-Small-Cell Lung Cancer. Pharmacol. Res..

[96] Zhou G., Zhang F., Guo Y., Huang J., Xie Y., Yue S., Chen M., Jiang H., Li M. (2017). MiR-200c Enhances Sensitivity of Drug-Resistant Non-Small Cell Lung Cancer to Gefitinib by Suppression of PI3K/Akt Signaling Pathway and Inhibites Cell Migration via Targeting ZEB1. Biomed. Pharmacother..

[97] Lin C.-C., Wu C.-Y., Tseng J.T., Hung C.-H., Wu S.-Y., Huang Y.-T., Chang W.-Y., Su P.-L., Su W.-C. (2021). Extracellular Vesicle MiR-200c Enhances Gefitinib Sensitivity in Heterogeneous EGFR-Mutant NSCLC. Biomedicines.

[98] Hu F.-Y., Cao X.-N., Xu Q.-Z., Deng Y., Lai S.-Y., Ma J., Hu J.-B. (2016). MiR-124 Modulates Gefitinib Resistance through SNAI2 and STAT3 in Non-Small Cell Lung Cancer. J. Huazhong Univ. Sci. Technolog. Med. Sci..

[99] Yu F., Liu J.-B., Wu Z.-J., Xie W.-T., Zhong X.-J., Hou L.-K., Wu W., Lu H.-M., Jiang X.-H., Jiang J.-J. (2018). Tumor Suppressive MicroRNA-124a Inhibits Stemness and Enhances Gefitinib Sensitivity of Non-Small Cell Lung Cancer Cells by Targeting Ubiquitin-Specific Protease 14. Cancer Lett..

[100] Fan D., Yang Y., Zhang W. (2022). A Novel Circ_MACF1/MiR-942-5p/TGFBR2 Axis Regulates the Functional Behaviors and Drug Sensitivity in Gefitinib-Resistant Non-Small Cell Lung Cancer Cells. BMC Pulm. Med..

[101] Zhang W., Dong Y.-Z., Du X., Peng X.-N., Shen Q.-M. (2019). MiRNA-153-3p Promotes Gefitinib-Sensitivity in Non-Small Cell Lung Cancer by Inhibiting ATG5 Expression and Autophagy. Eur. Rev. Med. Pharmacol. Sci..

[102] Liao J., Lin J., Lin D., Zou C., Kurata J., Lin R., He Z., Su Y. (2017). Down-Regulation of MiR-214 Reverses Erlotinib Resistance in Non-Small-Cell Lung Cancer through up-Regulating LHX6 Expression. Sci. Rep..

[103] Vadla G.P., Daghat B., Patterson N., Ahmad V., Perez G., Garcia A., Manjunath Y., Kaifi J.T., Li G., Chabu C.Y. (2022). Combining Plasma Extracellular Vesicle Let-7b-5p, MiR-184 and Circulating MiR-22-3p Levels for NSCLC Diagnosis and Drug Resistance Prediction. Sci. Rep..

[104] Li X., Chen C., Wang Z., Liu J., Sun W., Shen K., Lv Y., Zhu S., Zhan P., Lv T. (2021). Elevated Exosome-Derived MiRNAs Predict Osimertinib Resistance in Non-Small Cell Lung Cancer. Cancer Cell. Int..

[105] Delaney G.P., Barton M.B. (2015). Evidence-Based Estimates of the Demand for Radiotherapy. Clin. Oncol..

[106] Hayman T.J., Glazer P.M. (2021). Regulation of the Cell-Intrinsic DNA Damage Response by the Innate Immune Machinery. Int. J. Mol. Sci..

[107] Castle K.D., Kirsch D.G. (2019). Establishing the Impact of Vascular Damage on Tumor Response to High-Dose Radiation Therapy. Cancer Res..

[108] Chen X., Xu Y., Jiang L., Tan Q. (2021). MiRNA-218-5p Increases Cell Sensitivity by Inhibiting PRKDC Activity in Radiation-Resistant Lung Carcinoma Cells. Thorac. Cancer.

[109] Wang X.-C., Du L.-Q., Tian L.-L., Wu H.-L., Jiang X.-Y., Zhang H., Li D.-G., Wang Y.-Y., Wu H.-Y., She Y. (2011). Expression and Function of MiRNA in Postoperative Radiotherapy Sensitive and Resistant Patients of Non-Small Cell Lung Cancer. Lung Cancer.

[110] Li L., Liu H., Du L., Xi P., Wang Q., Li Y., Liu D. (2018). MiR-449a Suppresses LDHA-Mediated Glycolysis to Enhance the Sensitivity of Non-Small Cell Lung Cancer Cells to Ionizing Radiation. Oncol. Res..

[111] Jin Y., Su Z., Sheng H., Li K., Yang B., Li S. (2021). Circ_0086720 Knockdown Strengthens the Radiosensitivity of Non-Small Cell Lung Cancer via Mediating the MiR-375/SPIN1 Axis. Neoplasma.

[112] Qin P., Li Y., Liu J., Wang N. (2020). Knockdown of LINC00473 Promotes Radiosensitivity of Non-Small Cell Lung Cancer Cells via Sponging MiR-513a-3p. Free Radic. Res..

[113] Chen L., Ren P., Zhang Y., Gong B., Yu D., Sun X. (2020). Long Non-coding RNA GAS5 Increases the Radiosensitivity of A549 Cells through Interaction with the MiR-21/PTEN/Akt Axis. Oncol. Rep..

[114] Jiang L.-P., He C.-Y., Zhu Z.-T. (2017). Role of MicroRNA-21 in Radiosensitivity in Non-Small Cell Lung Cancer Cells by Targeting PDCD4 Gene. Oncotarget.

[115] Jiang S., Wang R., Yan H., Jin L., Dou X., Chen D. (2016). MicroRNA-21 Modulates Radiation Resistance through Upregulation of Hypoxia-Inducible Factor-1α-Promoted Glycolysis in Non-Small Cell Lung Cancer Cells. Mol. Med. Rep..

[116] Ma Y., Xia H., Liu Y., Li M. (2014). Silencing MiR-21 Sensitizes Non-Small Cell Lung Cancer A549 Cells to Ionizing Radiation through Inhibition of PI3K/Akt. Biomed Res. Int..

[117] Wang X., Wang W., Zhang Z.-B., Zhao J., Tan X.-G., Luo J.-C. (2013). Overexpression of MiRNA-21 Promotes Radiation-Resistance of Non-Small Cell Lung Cancer. Radiat. Oncol..

[118] He Z., Liu Y., Xiao B., Qian X. (2015). MiR-25 Modulates NSCLC Cell Radio-Sensitivity through Directly Inhibiting BTG2 Expression. Biochem. Biophys. Res. Commun..

[119] McDermott M., Eustace A.J., Busschots S., Breen L., Crown J., Clynes M., O’Donovan N., Stordal B. (2014). In Vitro Development of Chemotherapy and Targeted Therapy Drug-Resistant Cancer Cell Lines: A Practical Guide with Case Studies. Front. Oncol..

[120] Hafner M., Niepel M., Chung M., Sorger P.K. (2016). Growth Rate Inhibition Metrics Correct for Confounders in Measuring Sensitivity to Cancer Drugs. Nat. Methods.

[121] Pribluda A., de la Cruz C.C., Jackson E.L. (2015). Intratumoral Heterogeneity: From Diversity Comes Resistance. Clin. Cancer Res..

[122] Guarize J., Bianchi F., Marino E., Belloni E., Vecchi M., Donghi S., Lo Iacono G., Casadio C., Cuttano R., Barberis M. (2018). MicroRNA Expression Profile in Primary Lung Cancer Cells Lines Obtained by Endobronchial Ultrasound Transbronchial Needle Aspiration. J. Thorac. Dis..

[123] Roscilli G., De Vitis C., Ferrara F.F., Noto A., Cherubini E., Ricci A., Mariotta S., Giarnieri E., Giovagnoli M.R., Torrisi M.R. (2016). Human Lung Adenocarcinoma Cell Cultures Derived from Malignant Pleural Effusions as Model System to Predict Patients Chemosensitivity. J. Transl. Med..

[124] Liu X., Krawczyk E., Suprynowicz F.A., Palechor-Ceron N., Yuan H., Dakic A., Simic V., Zheng Y.-L., Sripadhan P., Chen C. (2017). Conditional Reprogramming and Long-Term Expansion of Normal and Tumor Cells from Human Biospecimens. Nat. Protoc..

[125] Kodack D.P., Farago A.F., Dastur A., Held M.A., Dardaei L., Friboulet L., von Flotow F., Damon L.J., Lee D., Parks M. (2017). Primary Patient-Derived Cancer Cells and Their Potential for Personalized Cancer Patient Care. Cell. Rep..

[126] Barretina J., Caponigro G., Stransky N., Venkatesan K., Margolin A.A., Kim S., Wilson C.J., Lehár J., Kryukov G.V., Sonkin D. (2012). The Cancer Cell Line Encyclopedia Enables Predictive Modelling of Anticancer Drug Sensitivity. Nature.

[127] Drost J., Clevers H. (2018). Organoids in Cancer Research. Nat. Rev. Cancer.

[128] Khan A.A., Betel D., Miller M.L., Sander C., Leslie C.S., Marks D.S. (2009). Transfection of Small RNAs Globally Perturbs Gene Regulation by Endogenous MicroRNAs. Nat. Biotechnol..

[129] Thomson D.W., Bracken C.P., Goodall G.J. (2011). Experimental Strategies for MicroRNA Target Identification. Nucleic Acids Res..

[130] Krützfeldt J., Rajewsky N., Braich R., Rajeev K.G., Tuschl T., Manoharan M., Stoffel M. (2005). Silencing of MicroRNAs in Vivo with “Antagomirs”. Nature.

[131] Ebert M.S., Sharp P.A. (2010). MicroRNA Sponges: Progress and Possibilities. RNA.

[132] Aquino-Jarquin G. (2017). Emerging Role of CRISPR/Cas9 Technology for MicroRNAs Editing in Cancer Research. Cancer Res..

[133] Lee I., Ajay S.S., Yook J.I., Kim H.S., Hong S.H., Kim N.H., Dhanasekaran S.M., Chinnaiyan A.M., Athey B.D. (2009). New Class of MicroRNA Targets Containing Simultaneous 5′-UTR and 3′-UTR Interaction Sites. Genome Res..

[134] Hausser J., Syed A.P., Bilen B., Zavolan M. (2013). Analysis of CDS-Located MiRNA Target Sites Suggests That They Can Effectively Inhibit Translation. Genome Res..

[135] Mayr C., Bartel D.P. (2009). Widespread Shortening of 3′UTRs by Alternative Cleavage and Polyadenylation Activates Oncogenes in Cancer Cells. Cell.

[136] Messina A., Langlet F., Chachlaki K., Roa J., Rasika S., Jouy N., Gallet S., Gaytan F., Parkash J., Tena-Sempere M. (2016). A MicroRNA Switch Regulates the Rise in Hypothalamic GnRH Production before Puberty. Nat. Neurosci..

[137] Gengenbacher N., Singhal M., Augustin H.G. (2017). Preclinical Mouse Solid Tumour Models: Status Quo, Challenges and Perspectives. Nat. Rev. Cancer.

[138] Rottenberg S., Borst P. (2012). Drug Resistance in the Mouse Cancer Clinic. Drug Resist. Updat..

[139] Hidalgo M., Amant F., Biankin A.V., Budinská E., Byrne A.T., Caldas C., Clarke R.B., de Jong S., Jonkers J., Mælandsmo G.M. (2014). Patient-Derived Xenograft Models: An Emerging Platform for Translational Cancer Research. Cancer Discov..

[140] Gómez-Cuadrado L., Tracey N., Ma R., Qian B., Brunton V.G. (2017). Mouse Models of Metastasis: Progress and Prospects. Dis. Model. Mech..

[141] Romani P., Nirchio N., Arboit M., Barbieri V., Tosi A., Michielin F., Shibuya S., Benoist T., Wu D., Hindmarch C.C.T. (2022). Mitochondrial Fission Links ECM Mechanotransduction to Metabolic Redox Homeostasis and Metastatic Chemotherapy Resistance. Nat. Cell Biol..

[142] Sanmamed M.F., Chester C., Melero I., Kohrt H. (2016). Defining the Optimal Murine Models to Investigate Immune Checkpoint Blockers and Their Combination with Other Immunotherapies. Ann. Oncol..

[143] de Rie D., Abugessaisa I., Alam T., Arner E., Arner P., Ashoor H., Åström G., Babina M., Bertin N., Burroughs A.M. (2017). An Integrated Expression Atlas of MiRNAs and Their Promoters in Human and Mouse. Nat. Biotechnol..

[144] De La Rochere P., Guil-Luna S., Decaudin D., Azar G., Sidhu S.S., Piaggio E. (2018). Humanized Mice for the Study of Immuno-Oncology. Trends Immunol..

[145] Montani F., Marzi M.J., Dezi F., Dama E., Carletti R.M., Bonizzi G., Bertolotti R., Bellomi M., Rampinelli C., Maisonneuve P. (2015). MiR-Test: A Blood Test for Lung Cancer Early Detection. J. Natl. Cancer Inst..

[146] Dama E., Melocchi V., Mazzarelli F., Colangelo T., Cuttano R., Candia L.D., Ferretti G.M., Taurchini M., Graziano P., Bianchi F. (2020). Non-Coding RNAs as Prognostic Biomarkers: A Mirna Signature Specific for Aggressive Early-Stage Lung Adenocarcinomas. Non-Coding RNA.

[147] Salgia R., Kulkarni P. (2018). The Genetic/Non-Genetic Duality of Drug “Resistance” in Cancer. Trends Cancer.

[148] de Bruin E.C., McGranahan N., Mitter R., Salm M., Wedge D.C., Yates L., Jamal-Hanjani M., Shafi S., Murugaesu N., Rowan A.J. (2014). Spatial and Temporal Diversity in Genomic Instability Processes Defines Lung Cancer Evolution. Science.

[149] Zhang J., Fujimoto J., Wedge D.C., Song X., Seth S., Chow C.W., Cao Y., Gumbs C., Gold K.A., Kalhor N. (2014). Intratumor Heterogeneity in Localized Lung Adenocarcinomas Delineated by Multiregion Sequencing. Science.

[150] Jamal-Hanjani M., Wilson G.A., McGranahan N., Birkbak N.J., Watkins T.B.K., Veeriah S., Shafi S., Johnson D.H., Mitter R., Rosenthal R. (2017). Tracking the Evolution of Non–Small-Cell Lung Cancer. N. Engl. J. Med..

[151] Aran D., Sirota M., Butte A.J. (2015). Systematic Pan-Cancer Analysis of Tumour Purity. Nat. Commun..

[152] Barkley D., Moncada R., Pour M., Liberman D.A., Dryg I., Werba G., Wang W., Baron M., Rao A., Xia B. (2022). Cancer Cell States Recur across Tumor Types and Form Specific Interactions with the Tumor Microenvironment. Nat. Genet..

[153] Cazet A.S., Hui M.N., Elsworth B.L., Wu S.Z., Roden D., Chan C.-L., Skhinas J.N., Collot R., Yang J., Harvey K. (2018). Targeting Stromal Remodeling and Cancer Stem Cell Plasticity Overcomes Chemoresistance in Triple Negative Breast Cancer. Nat. Commun..

[154] Dirkse A., Golebiewska A., Buder T., Nazarov P.V., Muller A., Poovathingal S., Brons N.H.C., Leite S., Sauvageot N., Sarkisjan D. (2019). Stem Cell-Associated Heterogeneity in Glioblastoma Results from Intrinsic Tumor Plasticity Shaped by the Microenvironment. Nat. Commun..

[155] Puram S.V., Tirosh I., Parikh A.S., Patel A.P., Yizhak K., Gillespie S., Rodman C., Luo C.L., Mroz E.A., Emerick K.S. (2017). Single-Cell Transcriptomic Analysis of Primary and Metastatic Tumor Ecosystems in Head and Neck Cancer. Cell.

[156] Hirsch F.R., Scagliotti G.V., Mulshine J.L., Kwon R., Curran W.J., Wu Y.-L., Paz-Ares L. (2017). Lung Cancer: Current Therapies and New Targeted Treatments. Lancet.

[157] Esposito M., Ganesan S., Kang Y. (2021). Emerging Strategies for Treating Metastasis. Nat. Cancer.

[158] Biswas A.K., Han S., Tai Y., Ma W., Coker C., Quinn S.A., Shakri A.R., Zhong T.J., Scholze H., Lagos G.G. (2022). Targeting S100A9-ALDH1A1-Retinoic Acid Signaling to Suppress Brain Relapse in EGFR-Mutant Lung Cancer. Cancer Discov..

[159] Rinaldi G., Pranzini E., Van Elsen J., Broekaert D., Funk C.M., Planque M., Doglioni G., Altea-Manzano P., Rossi M., Geldhof V. (2021). In Vivo Evidence for Serine Biosynthesis-Defined Sensitivity of Lung Metastasis, but Not of Primary Breast Tumors, to MTORC1 Inhibition. Mol. Cell.

[160] Herbst R.S., Morgensztern D., Boshoff C. (2018). The Biology and Management of Non-Small Cell Lung Cancer. Nature.

[161] Shukuya T., Ghai V., Amann J.M., Okimoto T., Shilo K., Kim T.-K., Wang K., Carbone D.P. (2020). Circulating MicroRNAs and Extracellular Vesicle-Containing MicroRNAs as Response Biomarkers of Anti-Programmed Cell Death Protein 1 or Programmed Death-Ligand 1 Therapy in NSCLC. J. Thorac. Oncol..

[162] Peng X.-X., Yu R., Wu X., Wu S.-Y., Pi C., Chen Z.-H., Zhang X.-C., Gao C.-Y., Shao Y.W., Liu L. (2020). Correlation of Plasma Exosomal MicroRNAs with the Efficacy of Immunotherapy in EGFR/ALK Wild-Type Advanced Non-Small Cell Lung Cancer. J. Immunother. Cancer.

[163] Boeri M., Milione M., Proto C., Signorelli D., Lo Russo G., Galeone C., Verri C., Mensah M., Centonze G., Martinetti A. (2019). Circulating MiRNAs and PD-L1 Tumor Expression Are Associated with Survival in Advanced NSCLC Patients Treated with Immunotherapy: A Prospective Study. Clin. Cancer Res..

[164] Fan J., Yin Z., Xu J., Wu F., Huang Q., Yang L., Jin Y., Yang G. (2020). Circulating MicroRNAs Predict the Response to Anti-PD-1 Therapy in Non-Small Cell Lung Cancer. Genomics.

[165] Halvorsen A.R., Sandhu V., Sprauten M., Flote V.G., Kure E.H., Brustugun O.T., Helland Å. (2018). Circulating MicroRNAs Associated with Prolonged Overall Survival in Lung Cancer Patients Treated with Nivolumab. Acta Oncol..

[166] Rajakumar T., Horos R., Jehn J., Schenz J., Muley T., Pelea O., Hofmann S., Kittner P., Kahraman M., Heuvelman M. (2022). A Blood-Based MiRNA Signature with Prognostic Value for Overall Survival in Advanced Stage Non-Small Cell Lung Cancer Treated with Immunotherapy. NPJ Precis. Oncol..

[167] Chaft J.E., Shyr Y., Sepesi B., Forde P.M. (2022). Preoperative and Postoperative Systemic Therapy for Operable Non-Small-Cell Lung Cancer. J. Clin. Oncol..

