# Supplementary material for: MicroRNAs and Drug Resistance in Non-Small Cell Lung Cancer: Where Are We Now and Where Are We Going
Source: Cancers (Basel). 2022 Nov 22;14(23):5731. doi: 10.3390/cancers14235731 (PMC9740066; doi:10.3390/cancers14235731)
Supplement: Supplementary file 1 [file cancers-14-05731-s001.zip › Supplementary Methods.pdf]

METHODS

miRNA annotation

To annotate miRNAs with a functional role in drug resistance in NSCLC, we performed a literature search using the PUBMED database with the following query-string: "miRNA AND NSCLC AND (resistance OR sensitivity) AND (Targeted therapy OR Immunotherapy OR Chemotherapy OR Radiotherapy OR all drug name currently used in clinics) NOT (Review[Publication Type])" with English language filter. The list of the drugs used in clinics were obtained from the National Comprehensive Cancer Network (NCNN) Non-small Cell Lung Cancer guidelines (NSCLC) Version 3.2022 (Supplementary Table 1). Publications were selected only if they contained miRNAs that *i)* showed differential expression between drug resistant and sensitive cancer cells (or tissues), and *ii)* miRNAs whose inhibition or enhanced expression resulted in a change of drug sensitivity profile. The nomenclature of miRNAs was manually curated using miRBase database (https://www.mirbase.org) with the information retrieved from each publication (e.g., real-time PCR primers for miRNA quantification, analysis report of prediction tool such as Target Scan Analysis or referring to miRBase previous ID).

Analysis of miRNAs associated with therapy response in NSCLC (referred to Fig. 1A)

To retrieved the number of miRNAs associated to a particular drug based on their evidence score, we first used a filter in column A ("Drug name") and then in column E ("Evidence score") of the Supplementary Table 2. The category of each drug was indicated in the figure based on the information reported in Supplementary Table 1. To obtain information about the trend of expression of selected miRNAs in resistant samples, we used the filter in column C ("regulation") in Supplementary Table 2. To test any changes in the global trend of miRNA expression for each drug, we compared the observed distribution of up/down-regulated miRNAs to an expected one of 50% (e.g half of miRNAs are downregulated and the other half is upregulated in resistant samples) by performing binomial test using Prism Software.
